# Supplementary material for: Patterns of Intron Gain and Loss in Fungi
Source: PLoS Biol. 2004 Nov 30;2(12):e422. doi: 10.1371/journal.pbio.0020422 (PMC532390; doi:10.1371/journal.pbio.0020422)
Supplement: Table S1 — Also available at http://genes.mit.edu/NielsenEtAl/. (4.3 MB ZIP). [file pbio.0020422.st001.zip › NielsenEtAl/html/399.html]

AN0579.1.NCU07719.1.MG07125.1.FG09722.1


```
 CLUSTAL W (1.82) Multiple Sequence Alignments - Introns Inserted


Sequence 1: NCU07719.1	255 aa
Sequence 2: MG07125.1	254 aa
Sequence 3: FG09722.1	253 aa
Sequence 4: AN0579.1	268 aa
Alignment Length: 272 aa
Number Identitical Residues: 160 aa
Alignment Score (without introns) 7441


MG07125.1 	MSSTTTT-TAQPITAETILSLFPDIDT----------------SGEALDGHDEEQIRLMD
NCU07719.1	MSTATTTNVAEPITAETILRLFPDIDT----------------RSDALEGHDEEQIRLMD
FG09722.1 	MSTTTTT-TNQPITAESMLRLFPDIDT----------------SSAPLSGHDEEQIRLMD
AN0579.1  	MSATETI---TRITADNVADIFPDVDTSLAREVLPQATTTSVANSNDLAGYDEEQVRLMD
          	**:: *      ***:.:  :***:**: : .  ..::::: : .  * *:****:****

MG07125.1 	EVCIVVDENDMPIGKGSKKIC1HLMTNIEKGLLHRAFSVFLFNDKNELLLQQRATEKITF
NCU07719.1	EVCIVTDQNDLPIGTASKKLC1HLMTNIDKGLLHRAFSVFLFNDKNELLLQQRATEKITF
FG09722.1 	EVCIVLDEDDKPIGTASKKIC1HLMTNIDKGLLHRAFSVFLFNDKNELLLQQRATEKITF
AN0579.1  	EVCIVLDDDDKPIGSASKKTC1HLMTNIDRGLLHRAFSVFLFDSQNRLLLQQRASEKITF
          	***** *::* ***..*** * ******::************:.:*.*******:*****

MG07125.1 	PDMWTNTCCSHPLGIPGETGSNLPDSIMGVKRAAQRKLDHELGIKKEQVPLENFHFLTRI
NCU07719.1	PDMWTNTCCSHPLNLANETGSTLPDAIAGVKNAARRKLDHELGIKQEQVPFEDFRFLTRI
FG09722.1 	PDMWTNTCCSHPLHIPTETGSTLEDSIAGVKRAAQRKLEHELGIKKEQVPFEDFHFLTRI
AN0579.1  	PDMWTNTCCSHPLGIPGETGSQLDAAILGVKRAAQRKLNHELGIKPEEVPIEKFEFFTRI
          	************* :. **** *  :* ***.**:***:****** *:**:*.*.*:***

MG07125.1 	HYKAPSDGKWGEHEV1DYILFIKANVDLDINKNEVQATQYTSADGLKAFFKDPAAKFTPW
NCU07719.1	HYKAPSDGKWGEHEI1DYILFIKANPEVNANLNEVQATQWVSAEKLKEMFKDPSLKFTPW
FG09722.1 	HYKAPSDGMWGEHEI1DYILFIKANVDLDINKNEVRDTQYVTPESLKQQFDDPSLVFTPW
AN0579.1  	HYKAPSDGKWGEHEI1DYILFIQADVVLEPNLNEVRDTRYVSADELKEMFKQTNLKFTPW
          	******** *****: ******:*:  :: * ***: *::.:.: **  *.:.   ****

MG07125.1 	FKLICESMLFEWWQHLDS-GLEKYTNEQGIRRML
NCU07719.1	FKLICESMLFEWWASLDN-GLEKYTDEQEIRRML
FG09722.1 	FKLICNSMLFEWWQNLDS-GLDKYLNEQEIRRM-
AN0579.1  	FKLICNSMLFEWWSHLGSPSLDQYKGETQIRRM-
          	*****:*******  *....*::* .*  ****
```
